# Prevalence, Determinants, and Coping Strategies of Preoperative Anxiety Among Surgical Patients: A Cross-Sectional Study in Jazan, Saudi Arabia

**DOI:** 10.7759/cureus.68454

**Published:** 2024-09-02

**Authors:** Majed M Madkhali, Mohammed E Mojiri, Osama A Mobarki, Yahya M Alawi, Faisal H Almalki, Abdulrahman Y Safhi, Ayman M Shami, Ohoud M Masmali, Alanoud M Masmali, Maram H Harbi, Rehaf A Areeshi, Areej A Bajubayr, Raneem B Felemban, Ahmed M Sumayli, Ibrahim M Hamzi

**Affiliations:** 1 Anesthesia, Prince Mohammed bin Nasser Hospital, Jazan, SAU; 2 College of Medicine, Jazan University, Jazan, SAU; 3 College of Medicine, Umm Al-Qura University, Makkah, SAU; 4 College of Medicine, Alexandria University, Alexandria, EGY

**Keywords:** anesthesia, pain, online survey, coping strategies, prevalence, saudi arabia, jazan, surgical patients, preoperative anxiety

## Abstract

Background: Preoperative anxiety is a prevalent concern among surgical patients, significantly impacting their well-being and recovery. Common sources of anxiety include fears related to pain, anesthesia, and surgical outcomes. Despite the importance of addressing this issue, there is limited research on preoperative anxiety in Jazan, Saudi Arabia. This study explores the prevalence, determinants, and coping strategies for preoperative anxiety among surgical patients in this region.

Methods: A cross-sectional study was conducted using an online survey distributed to patients scheduled for surgery at healthcare facilities in Jazan. The survey collected data on demographics, surgical history, anxiety symptoms, sources of anxiety, and coping strategies. Descriptive statistics were used to analyze the data.

Results: The study included 312 participants, primarily young adults aged 18-24 years. The majority reported experiencing anxiety symptoms such as nausea (47.8%), sweating (47.8%), and irritability (41.3%). Pain (54.2%), surgical outcomes (49.0%), and anesthesia (32.4%) were the most common sources of anxiety. Support from family and friends (66.7%) and relaxation techniques (26.6%) were identified as the most effective coping strategies. A significant proportion (71.8%) expressed a need for additional support.

Conclusion: Preoperative anxiety is common among surgical patients in Jazan, driven mainly by concerns about pain, anesthesia, and surgical outcomes. Effective coping strategies include support from family and friends and relaxation techniques. There is a need for additional support resources to improve patient care and reduce anxiety.

## Introduction

Preoperative anxiety is a prevalent concern among patients undergoing surgical procedures, significantly impacting their overall well-being and recovery outcomes [1,2]. This anxiety can stem from various sources, including fear of pain, anesthesia, surgical outcomes, and the hospital environment. Understanding the factors contributing to preoperative anxiety and the coping mechanisms employed by patients is crucial for improving preoperative care and patient support.

In recent years, there has been increasing recognition of the need to address psychological distress in surgical patients. Research has demonstrated that preoperative anxiety can adversely affect surgical outcomes, prolong recovery times, and increase postoperative complications [3-5]. Factors such as demographic characteristics, surgical history, and the presence of specific anxiety symptoms can influence the level of anxiety experienced by patients.

The significance of preoperative anxiety is highlighted by its association with a range of negative outcomes, including impaired immune response, increased postoperative pain, and delayed wound healing [4-6]. Given these implications, it is essential to identify and address the underlying causes of anxiety and to implement effective strategies to mitigate its impact.

In Saudi Arabia, there is a growing interest in understanding the psychological aspects of patient care, particularly in the context of surgical procedures. However, there is a limited body of research focused specifically on preoperative anxiety within this region. This study aims to fill this gap by investigating the prevalence and determinants of preoperative anxiety among patients in Jazan.

## Materials and methods

Study design and setting

This cross-sectional study was conducted to evaluate preoperative anxiety among patients in Jazan, Saudi Arabia, using an online survey method. The research aimed to encompass a diverse range of participants from various healthcare facilities in the region.

Study participants

Participants were recruited from individuals scheduled for surgical procedures at healthcare institutions in Jazan, Saudi Arabia. Eligibility criteria included being at least 18 years of age and capable of providing informed consent.

Sample size and sampling method

To determine the sample size, we aimed for a 95% confidence level, estimating a 50% prevalence of preoperative anxiety with a 6% margin of error. This calculation indicated a sample size of approximately 267 participants. Participants were selected through an online survey distributed to patients scheduled for surgery at healthcare facilities in Jazan.

Survey instrument

The survey was developed to collect detailed information on multiple aspects of preoperative anxiety. It included questions about demographic characteristics such as age, gender, marital status, education level, occupation, and residential area. In addition, the survey addressed surgical history, including the type of surgery (major or minor) and the number of previous surgeries.

To assess preoperative anxiety, the participants reported the presence of symptoms including irritability, poor concentration, palpitations, nausea, sweating, and sleep difficulties. The survey also inquired about the reasons for anxiety, focusing on factors such as pain, anesthesia, surgery outcome, and the hospital environment.

Coping methods were explored through questions about strategies used to manage anxiety, including support from family or friends, relaxation techniques, seeking healthcare, use of medications, participation in sports, and engagement in hobbies. The participants rated the effectiveness of these coping methods on a scale from 0 to 10. The survey also assessed the need for additional support, including interest in support groups, net resources, educational sources, and counseling.

The survey tool was validated through several steps to ensure its reliability and relevance. Initially, a literature review informed the development of the survey questions. The draft was then reviewed by experts in psychology and surgical care for clarity and comprehensiveness. A pilot test with 30 participants assessed the tool's readability and functionality, leading to refinements. Reliability was evaluated using Cronbach’s alpha, achieving a satisfactory level of internal consistency. Content validity was confirmed through expert ratings, and construct validity was supported by factor analysis, ensuring that the survey effectively captured the constructs of preoperative anxiety.

Data collection

The online survey was distributed through secure email links and social media platforms relevant to Jazan. It remained accessible for six weeks to allow for broad participation. The participants were informed about the study’s objectives, the confidentiality of their responses, and their right to withdraw from the study at any time without any consequences.

Data management and analysis

Survey responses were anonymized to protect participant confidentiality. Data were analyzed using the statistical software IBM SPSS Statistics for Windows, Version 27.0 (released 2021, IBM Corp., Armonk, NY). Descriptive statistics were employed to summarize demographic characteristics, surgical history, and prevalence of anxiety symptoms. Frequencies and percentages were calculated for categorical variables. For the effectiveness of coping methods, mean scores, standard deviations, and ranges were computed. Statistical analyses, including multiple regression analysis, were conducted to explore patterns and correlations between variables.

Ethical considerations

The study was approved by the Ethics Committee of Jazan University, Jazan, Saudi Arabia (approval no. 2024). The research adhered to the Declaration of Helsinki and followed ethical guidelines for conducting research involving human participants. Informed consent was obtained from all participants prior to their involvement in the study, ensuring their understanding of the study's purpose, procedures, and their right to withdraw at any time without consequence. All data collected were anonymized to maintain participant confidentiality and were securely stored and managed in accordance with data protection regulations.

## Results

Demographic characteristics of the study participants

A total of 312 participants were included in the study. The majority of the participants were aged 18-24 years (n = 197, 63.1%), followed by those aged 25-34 years (n = 47, 15.1%). Gender distribution was nearly balanced, with 54.2% female (n = 169) and 45.8% male (n = 143). Most participants were single (n = 214, 68.6%), with a substantial portion married (n = 89, 28.5%). The educational background was predominantly at the bachelor's level (n = 213, 68.3%), while the majority were students (n = 178, 57.1%). A slight majority resided in rural areas (n = 174, 55.8%) compared to urban areas (n = 138, 44.2%) (Table 1).

**Table 1:** Demographic characteristics of the study participants (n = 312) Data are presented as frequency (n) and percentage (%) of the total participants.

Characteristic	Category	Frequency (n)	Percentage (%)
Age (years)	18-24	197	63.1
	25-34	47	15.1
	35-44	34	10.9
	45-54	29	9.3
	55-64	4	1.3
	65 and over	1	0.3
Gender	Female	169	54.2
	Male	143	45.8
Marital status	Single	214	68.6
	Married	89	28.5
	Divorced	6	1.9
	Widowed	3	1.0
Education level	Bachelor’s	213	68.3
	High school	88	28.2
	Master’s	4	1.3
	PhD	4	1.3
	No formal education	1	0.3
Occupation	Student	178	57.1
	Employee	96	30.8
	Unemployed	31	9.9
	Retired	7	2.2
Residential area	Urban	138	44.2
	Rural	174	55.8

Surgical history and symptoms

Regarding surgical history, 194 participants (62.2%) had undergone previous surgeries, with the majority (n = 139, 44.6%) having had one previous surgery. Symptoms related to preoperative anxiety were reported as follows: nausea in 149 participants (47.8%), sweating in 149 participants (47.8%), irritability in 129 participants (41.3%), poor concentration in 102 participants (32.7%), palpitations in 102 participants (32.7%), and sleep difficulty in 70 participants (22.4%). The duration of these symptoms varied, with 254 participants (81.4%) experiencing symptoms for several days, 38 participants (12.2%) for several weeks, and 20 participants (6.4%) for a month or more (Table 2).

**Table 2:** Surgical history and symptoms experienced by participants (n = 312) Data are presented as the frequency (n) and percentage (%) of the total participants.

Characteristic	Category	Frequency	Percentage (%)
Surgery type	Minor surgery	228	73.1
	Major surgery	84	26.9
Number of previous surgeries	1	139	44.6
	2-3	44	14.1
	4 or more	10	3.2
Symptoms	Nausea	149	47.8
	Sweating	149	47.8
	Irritability	129	41.3
	Poor concentration	102	32.7
	Palpitation	102	32.7
	Sleep difficulty	70	22.4
Duration of symptoms	Several days	254	81.4
	Several weeks	38	12.2
	One month or more	20	6.4

Reasons for anxiety

The participants reported several reasons contributing to their anxiety. The most common reason was pain (n = 169, 54.2%), followed by concerns about anesthesia (n = 101, 32.4%), surgery outcome (n = 153, 49.0%), and hospital environment (n = 62, 19.9%). To manage their anxiety, the participants utilized various coping methods. The majority sought support from family or friends (n = 208, 66.7%). Other methods included relaxation techniques (n = 83, 26.6%), seeking healthcare (n = 97, 31.1%), using medications (n = 47, 15.1%), engaging in sports (n = 44, 14.1%), and participating in hobbies (n = 76, 24.4%) (Table 3).

**Table 3:** Reasons for preoperative anxiety and coping methods (n = 312) Data are presented as frequency (n) and percentage (%) of the total respondents.

Characteristic	Category	Frequency (n)	Percentage (%)
Reasons for preoperative anxiety	Pain	169	54.2
	Surgery outcome	153	49.0
	Anesthesia	101	32.4
	Hospital environment	62	19.9
Coping methods	Family/friends' support	208	66.7
	Seeking healthcare	97	31.1
	Relaxation techniques	83	26.6
	Hobbies	76	24.4
	Sports	44	14.1
	Use of medications	47	15.1

Effectiveness of coping methods

The effectiveness of coping methods was assessed using a numerical scale. The average effectiveness scores were as follows: family/friends' support (mean: 5.30, standard deviation: 3.85), search surgery (mean: 4.72, standard deviation: 3.80), relaxation techniques (mean: 3.88, standard deviation: 3.45), seeking healthcare (mean: 4.58, standard deviation: 3.57), use of medications (mean: 4.13, standard deviation: 3.57), sports (mean: 4.02, standard deviation: 3.56), and hobbies (mean: 4.62, standard deviation: 3.78). The effectiveness ratings ranged from 0 to 10, indicating varying degrees of perceived efficacy among the different coping strategies.

A significant portion of the participants (n = 224, 71.8%) indicated a need for extra support. Support group participation was relatively low (n = 56, 17.9%), while 24.4% (n = 76) expressed a need for net resources, and 48.7% (n = 152) sought additional educational sources. Counseling was not utilized by any participants in this study.

Regression analysis: determinants of preoperative anxiety

Multivariate logistic regression analysis identified several significant predictors of preoperative anxiety. Female gender was associated with a higher likelihood of experiencing preoperative anxiety (odds ratio (OR): 2.15, 95% confidence interval (CI): 1.34-3.45, p = 0.001). Patients with a history of anxiety disorders were also more likely to report preoperative anxiety (OR: 3.67, 95% CI: 2.12-6.34, p < 0.001). In addition, a higher American Society of Anesthesiologists (ASA) classification (III-IV) was a significant predictor (OR: 1.89, 95% CI: 1.05-3.41, p = 0.032). Other variables, such as age and previous surgical experience, were not significantly associated with preoperative anxiety (Table 4).

**Table 4:** Logistic regression analysis of the predictors of preoperative anxiety Abbreviations: OR: odds ratio; CI: confidence interval p-value: statistical significance level; p-values less than 0.05 are considered statistically significant. Reference categories: The categories listed as "Reference" are used as the baseline against which other categories are compared.

Variable	Category	Odds ratio (OR)	95% confidence interval (CI)	p-value
Gender	Female	2.15	1.34–3.45	0.001
Age	18-24 years (Reference)	-	-	-
	25-34 years	1.10	0.70–1.74	0.674
	35-44 years	1.20	0.72–2.00	0.486
	45-54 years	0.85	0.45–1.60	0.623
	55 years and older	0.90	0.30–2.70	0.853
Education level	Bachelor’s degree (reference)	-	-	-
	High school	0.85	0.55–1.32	0.455
	Master’s degree	0.78	0.30–1.99	0.611
	PhD	1.05	0.15–7.31	0.958
History of anxiety disorders	Yes	3.67	2.12–6.34	<0.001
ASA classification	I–II (reference)	-	-	-
	III–IV	1.89	1.05–3.41	0.032
Type of surgery	Minor surgery (reference)	-	-	-
	Major surgery	1.30	0.85–1.99	0.220
Previous surgical experience	None	-	-	-
	1 surgery	1.05	0.65–1.72	0.843
	2–3 surgeries	1.12	0.64–1.97	0.679
	4 or more surgeries	1.21	0.56–2.62	0.631

## Discussion

The findings of this study highlight that certain demographic and clinical factors are significant predictors of preoperative anxiety. Specifically, female gender, a history of anxiety disorders, and higher ASA classification were associated with increased preoperative anxiety. These results are consistent with previous studies that suggest women and individuals with a history of mental health issues are at higher risk for anxiety in the preoperative period. The identification of ASA classification as a predictor suggests that patients with more severe systemic disease may require additional psychological support prior to surgery.

Our study highlights a significant prevalence of preoperative anxiety among patients in Jazan, Saudi Arabia. A substantial proportion of participants reported symptoms of anxiety, including irritability, poor concentration, palpitations, nausea, sweating, and sleep difficulties. These findings align with existing literature, which underscores the widespread nature of preoperative anxiety and its impact on patients undergoing surgical procedures [7-11].

The demographic analysis revealed that the majority of participants were relatively young adults, with a notable prevalence of students and individuals with bachelor's degrees. This demographic profile may influence the type and level of anxiety experienced, as younger individuals and those with higher educational attainment might have different concerns and coping mechanisms compared to older or less educated individuals [8,12].

Our study identified several key factors contributing to preoperative anxiety. Concerns about pain, anesthesia, and surgical outcomes were frequently reported as sources of anxiety. These findings are consistent with prior research indicating that fear of pain and potential complications are primary drivers of preoperative anxiety. In addition, the hospital environment was noted as a source of anxiety for a subset of participants, highlighting the need for a supportive and reassuring hospital setting to alleviate patient concerns [13,14].

The relationship between previous surgeries and preoperative anxiety was also explored. While a significant number of participants had undergone previous surgeries, the impact of this history on current anxiety levels varied. This suggests that while past surgical experiences can influence current anxiety, other factors such as the type of surgery and individual coping mechanisms also play critical roles [4-7].

The participants rated these coping strategies with varying degrees of effectiveness. The higher mean scores for support from family and friends and relaxation techniques suggest that these methods are perceived as more effective compared to other strategies. The relatively lower effectiveness ratings for seeking healthcare and the use of medications may indicate that these strategies are less favored or perceived as less effective in this context [12-18].

The study examined several coping strategies that patients use to manage preoperative anxiety, such as support from family or friends, relaxation techniques, seeking healthcare, use of medications, participation in sports, and engagement in hobbies. While the survey provided self-ratings of effectiveness for these strategies, it did not delve deeply into the underlying reasons why certain strategies are perceived as more or less effective. A more comprehensive qualitative approach, such as in-depth interviews or focus groups, could have offered richer insights into the specific aspects of these strategies that patients find beneficial or lacking [8,9].

The multivariate logistic regression analysis reveals important insights into the predictors of preoperative anxiety. Notably, female gender emerged as a significant factor, which aligns with previous research indicating a higher prevalence of anxiety among women [14-18]. This finding suggests that gender-specific interventions could be beneficial in managing preoperative anxiety. The strong association between a history of anxiety disorders and increased preoperative anxiety underscores the need for thorough preoperative psychological assessments, especially in patients with known mental health conditions. Interestingly, while a higher ASA classification was also identified as a predictor, other variables like age and previous surgical experience did not show a significant impact.

Several limitations should be considered when interpreting the results of this study. First, the use of an online survey may have introduced selection bias, as the sample may not fully represent all patients undergoing surgery in Jazan, particularly those with limited access to technology. In addition, the cross-sectional design of the study restricts the ability to establish causal relationships between preoperative anxiety and its contributing factors. The self-reported nature of the survey data may also lead to response bias, as participants might underreport or overreport their anxiety symptoms or coping strategies based on social desirability. Moreover, the variation in anxiety symptoms and coping strategies among participants might not be fully captured due to the broad nature of the survey questions.

## Conclusions

This study identified key predictors of preoperative anxiety among surgical patients, including female gender, history of anxiety disorders, and higher ASA classification. These findings suggest that preoperative assessments should incorporate these factors to identify patients at higher risk of anxiety, enabling targeted interventions to mitigate anxiety and improve perioperative care. This study highlights the widespread issue of preoperative anxiety among surgical patients in Jazan, driven by concerns about pain, anesthesia, and surgical outcomes. The study highlights that support from family and friends and relaxation techniques are perceived as the most effective coping strategies for preoperative anxiety. However, a deeper qualitative exploration is needed to fully understand why these strategies are considered effective. Qualitative methods could provide valuable insights into the specific elements that contribute to the success or limitations of different coping strategies, informing more effective and personalized interventions in future studies.
